# Benefits and risks: Views of humanitarian organizations in Lebanon on corporate assistance

**DOI:** 10.1371/journal.pgph.0002291

**Published:** 2023-11-10

**Authors:** Jihad Makhoul, Catherine El Ashkar, Melissa Mialon, Anna Levy, Diana Sabbagh, Rima Nakkash

**Affiliations:** 1 Faculty of Health Sciences, Department of Health Promotion and Community Health, American University Beirut, Beirut, Lebanon; 2 Trinity Business School, Trinity College Dublin, Dublin, Ireland; 3 New York University, Robert F. Wagner Graduate School of Public Service, New York, New York, United States of America; 4 Global and Community Health Department, George Mason University, Fairfax, Virginia, United States of America; Royal Infirmary of Edinburgh and University of Edinburgh, UNITED KINGDOM

## Abstract

Compounding humanitarian and political crises within and across countries have been met with shrinking public resources for coordination, recovery, and mitigation. This resource constrained humanitarian environment presents opportunities for multinational corporations to supplement budgets and actively participate in new markets through connecting with humanitarian work. Given the well-established influence of corporations on public health, an assessment of industry funding to humanitarian assistance is necessary especially in the fragile context of Lebanon with a substantial refugee population and multiple compounding crises. This paper examines three aspects of corporate assistance in humanitarian crises in Lebanon. It investigates the modality of corporate assistance to humanitarian agencies, the extent to which humanitarian agency staff are aware of implications of this assistance along with any ethical considerations related to it, and both the risks and benefits for corporations and people. This study explores the views of 14 local and international humanitarian agencies in Lebanon) through in-depth interviews conducted between 2020 and 2022. Interviews were recorded, transcribed and subject to thematic analysis. All agencies participating in the study provided social and health assistance as well as education, vocational training, and other services to refugees or Lebanese. Findings indicate that the majority of them receive corporate funding in varying amounts and in-kind contributions to support various projects. Despite imposed conditions by the corporations, such as posting logos and stories, the agencies perceived the benefits of partnering, mentioning financial assistance in time of need, and flexible agendas that outweigh the risks of conflicts of interest of corporate branding on the populations they serve. Benefits to the corporations themselves relate to corporate social responsibility, increased market reach and visibility. Challenges in partnering with for-profits include ethical considerations and programmatic issues, however no guidelines were reported to exist to detect corporate conflicts of interest, instead most of the agencies rely on their value systems for screening.

## Introduction

Humanitarian organizations are major actors that respond to people affected by crises. Refugees and forcibly displaced populations are especially vulnerable to such crises; having to leave their homes, they often endure compounding issues in crossing borders including social and political violence, and find themselves relying on a combination of international organizations, diaspora networks, charities or community organizations for accessing basic services [1]. Precarious legal and social status exposes refugees and displaced people to exploitation, gender discrimination and other forms of abuse in the course of this process [2].

Humanitarian aid has been historically organized by and through international organizations—including multilateral organizations, such as United Nations agencies (e.g.: the World Food Program (WFP) and United Nations High Commissioner for Refugees (UNHCR)), bilateral and non-governmental organizations (NGO), such as Care International, Oxfam, and the International Federation of Red Cross-, religious institutions and organizations, philanthropic initiatives, and grassroots and community organizations in nearby as well as distant countries [3,4]. Sources of humanitarian funding come from small or large private individual donations, state budgets, and corporations, amongst others [5].

Local and global companies are major contributors to humanitarian aid globally through financial or in-kind contributions [6]. For example, in 2015, the UNHCR noted the key role of certain companies as “humanitarian innovators”, such as VISA (banking sector), Verizons (telecommunications sector), Google, or GSK (pharmaceutical sector) [7]. In 2012, financial contributions from private sources amounted to an estimated US$4.1 billion for humanitarian assistance, representing 24% of the total international response globally [8]. For example, CARE, an NGO based in the U.S., that reaches over 100 countries to address poverty and provides humanitarian aid programs, has had corporate sponsors such as Coca-Cola, the PepsiCo Foundation, Johnson and Johnson, Pfizer, Nike, Procter and Gamble, amongst others [9]. Corporate support to the development and health sector is key in the UN Sustainable Development Goals (SDG), with SDG 17 focusing on partnerships with the private sector [10]; the UN Global Compact is another UN instrument promoting corporations involvement in health and human right issues [11]. Launched in 2000, it is a platform for companies to voluntarily engage in the achievement of the SDG [12]. The World Humanitarian Summit in 2016 earned commitments from multilateral development banks and organizations to work more closely and regularly through public-private initiatives to resourcing humanitarian response, and the Syrian refugee crisis in particular [13]. The process of organizing and delivering relief services and goods began to receive significant corporate funding especially after the Indian Ocean Tsunami of 2004 [14–16].

Corporate funding is framed as having an added value in its ability to help humanitarian organizations do more work, integrate their work with others, maximize the advantage of collective action, improve performance, and save on expenses [17]. These corporate-humanitarian arrangements also have multiple advantages for corporations, such as benefits for tax reductions and avoidance; increasing faith in and trust of market-based solutions for health, economic, and governance issues [18]; access to key resources for marketing purposes such as new networks and customers [16,19]. Regulatory oversight and democratic debate over the costs and benefits of these arrangements are often considered secondary to the fast pace and overwhelming need for resources and expertise when confronting complex crises [16,20].

Although scarce, published research assessing corporate engagements with humanitarian organizations has been positive in general, and mostly limited to the technical aspects, such as coordination; corporate social responsibility; training and improving governance [21–23]. Meanwhile, other features of corporate funding related to corporations and humanitarian assistance, and the possible direct or indirect influence on the vulnerable populations being served, have not been scrutinized. Although the field of research on commercial determinants of health has grown over the last few decades, there has been very little done to specifically understand the process and impact of corporate assistance (including funding) to humanitarian organizations. Conflicts of interest and corporate interference in public policy, research and practice have been well documented for the pharmaceutical, tobacco, food and beverage, alcohol and arms industries [24–26]. In an era of shrinking financial resources, external funding from for-profit organizations has been framed as both necessary and inevitable. The political economy of incentives, immediate impacts, and long-term implications of these shifts on governance, health, equity, and democratic oversight among other factors are underexplored and not well understood. As the frequency, volume, and normalization of public-private financing and partnerships becomes a prominent feature of crisis response, and potential substitute for political commitments or arrangements required to resolve crisis causes and effects over time, this paper attempts to make a modest contribution toward advancing this discussion. Research exploring the experiences and views of humanitarian organizations *vis-à-vis* receiving corporate assistance is urgently needed for a better understanding of related risks and benefits, and preparing effective responses.

This study aimed to explore the experiences of humanitarian organizations in Lebanon with corporate donations, funding and other incentives (grouped under the term “corporate assistance”, interchangeably, hereafter, except when describing specific elements), associated challenges and perceived influences on the recipients of such assistance. We collected data between May 2020 and January 2022. The first, second and last authors are native of Lebanon and fluent in Arabic and English. The fourth author is a native English speaker, with experience living in the Middle East, and expertise on corporate assistance in the humanitarian space. All authors have expertise on the commercial determinants of health.

### Background information on the case country, Lebanon

Over the last fifty years, as settler colonialism, Occupation, authoritarianism, and conflict have triggered various migrations through the Levant, Lebanon has been a regular arrival for civilians from neighboring countries [27]. Today, Lebanon hosts the largest number of refugees per capita including Palestinian, Ethiopian, Sudanese, Iraqi, and Syrian [28]. There are currently 479,500 registered Palestinian refugees in Lebanon [29]. More recently, Lebanon hosted over 1.5 million Syrian refugees, hundreds of thousands of whom were unregistered [28]. The recent influx of Syrian refugees since 2011 placed considerable pressure on the already inadequate education, health, and economic infrastructure of the country [30]. In 2020, the COVID-19 pandemic, and then the August 2020 Beirut Port blast, exacerbated the situation in an already fragile state. The blast resulted in more than 200 deaths, 7000 injuries, and massive destruction and devastation across commercial and residential neighborhoods. reaching around 40% of Beirut, and severely damaging health infrastructure and essential food and medical supplies [31–33]. Since early 2021, there is also an absence of a government in Lebanon, and the country faces a compounding debt crisis [34]. All of these meant that there is a vacuum of resources and centralized governance in the country.

As a result, Lebanon witnessed a widespread humanitarian response in the aftermath of the Beirut blast destruction [33]. Lebanese civil society was mobilized alongside individual donors in the country and abroad, to send assistance to those affected and volunteer to help with cleanup and rehabilitation efforts, and to provide basic emergency items [35]. The UN initiated and coordinated an international humanitarian response to ensure assistance was provided. For example, a ‘Reform, Recovery, and Reconstruction Framework’ was initiated in 2020 by the European Union, the World Bank and the UN [35].

Amidst this backdrop, corporations have been a key source of funding for humanitarian aid in their provision of assistance in various forms in Lebanon. Examples included Nestlé, providing donations for the Middle East region in the form of food, beverage, water and medical supplies [36]; PepsiCo offering education scholarships to needy refugee youth in Lebanon through a local community organization [37], and Coca-Cola Middle East implementing the ’Eradication of Hunger by 2020’ initiative with the Food Banking Regional Network [38].

## Materials and methods

We followed a qualitative research approach, with in-depth interviews of a convenient purposive sample of representatives of non-governmental humanitarian organizations (“humanitarian organizations” hereafter) working in Lebanon. The organizations selected for our study provided any type of social and health assistance to refugees or Lebanese. A list of these organizations was compiled using: i) publicly available information–including the ‘Arab NGO Directory’ [39]; ii) announcements of job opportunities through emails from the Career Office at the Faculty of Health Sciences, American University of Beirut, Lebanon; iii) the ‘Daleel Madani’ civil society directory; and iv) snowball searches, where information about additional organizations was identified from the searches above. The final list of agencies which we contacted was composed of 79 humanitarian organizations.

The directors/program managers of the humanitarian organizations were then invited to participate in the study through their email or publicly available email addresses of the organizations, inquiring about the contact details of the director/program manager. The email had details about the aim of the study and asked for referral to individuals who were best suited for the interview. Out of the 79 humanitarian organizations that were contacted, 7 replied that they did not receive corporate funding, thus they were not eligible for participating, and 14 organizations agreed to participate, representing 4 international and 10 local organizations. Seven of the interviewees were general managers, directors or founders of the humanitarian organizations, while the other seven were senior programs associate; grants manager; operations manager; head of marketing and sponsorship; deputy head of missions for program; evidence learning and accountability manager; and external relations officer. Although their areas of work were diverse, the organizations converged in that they worked with refuge populations, mainly Syrian refugees or vulnerable populations including Lebanese youth, children, and elderly. Their activities centered on health, youth empowerment, vocational training, education, crime prevention, agriculture and environment, as well as rebuilding destroyed residences in Beirut—all spanning Beirut and disadvantaged areas in Lebanon.

The first and last authors conducted semi-structured interviews in both English and Arabic until we reached data saturation, as evidenced by the repetition of themes described below. We used an interview guide of open-ended questions that was tested in an earlier small-scale student project. The questions explored the humanitarian organizations’ work; sources of funding; the process for receiving funds or other incentives; perceived advantages and disadvantages of receiving incentives from corporations; conditions stipulated by corporations; and existing guidelines to regulate these interactions with corporations. After the first 4 interviews, the research team added a question to the interview guide pertaining to a hypothetical situation assessing their response to being approached by obvious health harming industries to provide financial assistance.

Thirteen out of the 14 interviews were held virtually through Zoom platform, and one was carried out in-person after participants signed a consent form. The interviews lasted between 20 and 90 minutes, and were digitally recorded with the consent of the participants. The interviews were then transcribed verbatim by the second author (in Arabic and English), subsequently coded and subject to thematic analysis–a process led by the first and second authors, involving identifying, analyzing and describing the data generated from the interviews and gradually deducing themes [40]. The researchers read the transcripts and coded them using an axial coding system in preparation for the next step of identifying emerging themes and patterns to answer the research question. The study was approved by the American University of Beirut Institutional Review Board on September 4, 2019 (approval number is SBS SBS-2019-0297-20190105). Given the qualitative nature of the methods, authors had access to information that could identify individual participants during the recruitment, the interview, and after data collection during the analysis phase. The identity of participants however is not revealed in the manuscript as agreed per the consent form. Participants are referred to by number and affiliation to local vs international organization.

## Results

### Sources and nature of corporate assistance

Our participants reported receiving funds in varying magnitudes from both corporations and other international sources, such as UN organizations, embassies, foreign development organizations, UN Office for the Coordination of Humanitarian Affairs and foreign aid (e.g., USAID, aid from the UK, etc.). The majority of the humanitarian organizations reported receiving money from for profit corporations, in varying amounts and in-kind contributions. For two organizations, funding came from the businesses of the organizations’ board members. Donors included private foundations linked to corporations, or directly from multinational companies such as Nestlé, PepsiCo, and Proctor & Gamble, or money from the local banking, automobile industry and telecommunications sector. Our participants also mentioned receiving a small sum of money from corporations through services from an advertising company, as well as in-kind donations, such as first aid kits, vouchers, cleaning materials, laptops, and so on.

Funds were used to support a myriad of projects. For example, funds from PepsiCo were allocated to rehabilitate restaurants damaged by the Beirut blast and to cover the tuition fees of university students. Procter & Gamble supported menstruation programs in schools, and provided toothbrushes and toothpaste for school students. Unilever (Lipton specifically) distributed its tea as part of a Ramadan package, while Phillip Morris distributed sanitizers during COVID-19 pandemic. Pharmaceutical companies were also mentioned as providing in kind donations for two agencies, such as blood glucose test strips for diabetes machines. One agency received funding from Pfizer for the rehabilitation of a hospital wing. Other sources of funding included governmental funding (Canadian source), crowdfunding and fundraising, international headquarters, other organizations; sponsorship programs; donations; and charity money.

### Process for applying and receiving corporate assistance

The process for receiving support from corporations, in particular, included two different modes: i) a proactive one where organizations seek out donors, apply to calls, or use their personal networks; and ii) a passive approach, where the organizations are approached by funders. The humanitarian organizations also communicate their need for funding through advertising or press releases, to showcase their work. About a third of organizations interviewed reported having communication departments that supported the proactive approach, which included conducting research to identify potential donors or reaching out to donors they have worked with before.

*“We raise funds through different approaches. We do our own research to identify potential donors, so we go to the websites of international agencies, UN agencies, banks, corporations. We reach out to previous partners or donors we worked with and we ask if there is a future cause we can work on. We also rely on the fact that we get approached by international NGOs looking for a local partner to implement a project on the ground.” (Participant 5, local agency)*

The more passive approach seems to be predominant among the more established and reputable organizations. Twelve of the organizations reported being approached by other organizations or corporations either through an open call for proposals to provide either in-kind donations or medicine, or cash. Four organizations were specifically approached after the Beirut Port blast. Board members were reported as being directly approached by corporations.

*“…after the blast, all of them (corporations), started mapping who were the NGO that were active, well-governed and transparent. We were only like three or four, we had great visibility after the blast. They saw our website and our application [to their call for funding] and called us and told us that we have been shortlisted and then they chose us. They met with us and asked to go with us to see our work. And then they decided to give us the grant.”(Participant 10, local agency)*

One humanitarian organization mentioned being linked to corporations through the UN Global Compact network for funding.

*“In the Global Compact we are working on the Agenda 2030, they are linking us with corporations because today, all companies care about CSR.” (Participant 14, local NGO)*

### Internal guidelines and principles for engagement

Only one participant reported that their organization has written guidelines for detecting and managing conflicts of interest (COI) and any undue influence that may result from corporate funding. Another two participants noted that their head offices or their Board of Trustees were responsible for deciding on funding sources. The other guidelines that four participants mentioned included details about COI for individual staff members, sexual harassment and anti-corruption. The majority of our participants reported that they implicitly adhered to a value system or agreed upon criteria when approached by corporations.

Unacceptable sources of funding included those whose values contradict those of the organizations, for example tobacco (n = 4 organizations who mentioned this), alcohol (n = 4), fast food industries (n = 2), political and religious groups (n = 2), or others mentioned once, such as the lottery industry, violators of human rights, child unfriendly, those associated with war crimes and money laundering, or ‘harm doers’. Other sources of funding which were also described as unwelcome include those which impose conditions (n = 2) and those whose scope of work does not align with theirs (n = 1).

Participants described the processes which they follow as part of the ‘homework’ that some of them do, such as vetting or screening for possible problems guided by a checklist, which a communications officer takes care of, or investigating the CEO’s reputation before memorandums of understanding were signed. Despite some reference to this type of investigation to assess the credibility and the mission of corporations, four of the participants reported that they were rather flexible and would accept financial support from any source because they were desperate for funds.

*“We go through a screening and vetting process to make sure that we are not at risk of tarnishing our reputation by keeping an eye out on the CEOs as an example of corporations we want to work with, to make sure the CEO’s reputation is good, and that includes checking and keeping an eye out from the beginning and during the partnership.” (Participant 2, international agency)*

*“The communications manager would look for sources guided by criteria and these sources need to be non-harmful for us or the children.” (Participant 11, local agency)*

*“Let me tell you the truth, these days I don’t think anyone will care about this, because even though they might drink, you know, drink Pepsi or use Maggi or buy or use Nestlé’s products, that’s fine because there is an added value. They are providing us with money. These days we have no choice, we need money to work and these corporations are there to help.” (Participant 9, local agency)*

### Conditions to receive assistance

Seven of our participants mentioned that when they received funds from corporations, they were asked to show the logos of the corporations, either on social media or on tangible products like printed materials, food packages, doors/entrances, trucks, classrooms; and this sometimes would be from corporate guidelines specifying where the logos should be placed.

*“… [the donors] asked for logo positioning in a certain location. In Ramadan, on the imsakiyyi) calendar for Ramadan with prayer times), on the bag, on the donated pack, we put the logo, on the website, on social media…. We put logo on pharmacy bags or prescription papers, depending on what printing material to be distributed to the patients, based on the money they pay.” (Participant 14, local agency)*

*“They asked us to put the logo, because they get the money from their country, and the private logo is important as it shows how they are spending their money. They trust us and we do what we need. And everyone is happy.” (Participant 9, local agency)*

Eight participants also mentioned other requirements, such as posting stories, videos, and testimonials from recipients, often on the social media accounts of both parties.

*“we made videos, and this was posted on social media and also on their website.” (Participant 9, local agency)*

*“(showing us social media pictures), especially as we have followers, our insights are very high, strategically, [using] digital [platforms] today is completely different for corporations than if they used [their] logos on a bag for example” (Participant 14, local agency)*.

*“our communications manager will send them stories that they can use about the change that the money has made, or that kind of thing. And then, that is important for us as well, reporting on the targets that we said that we were going to meet.” (Participant 1, international agency)*

In general, the conditions for getting a donation or other incentive from corporations seem to be agreed upon and not necessarily contested. However, three participants stated they did not necessarily comply with the conditions, or do so only if the conditions are appropriate and do not contradict their own organizational guidelines. For example, one organization refused to put the name of a company on a hospital wing, while another stated that if they accepted money from the alcohol, tobacco, or pharmaceutical sectors, they would insist on no visibility whatsoever for the company; and yet another participant said they did not accept to post photos or videos for the sake of confidentiality of the recipients.

*“..there was a person who approached us and said we will help you if you put the name of our company on a hospital wing for children, we will give you money. We felt this was too much and we did not accept.” (Participant 6, local agency)*

*“Two options, either we reject the idea as a whole because it is an alcohol or tobacco company, an unhealthy brand that we cannot promote or take money from. So we either say no. In case we say yes, there will be no visibility for them whatsoever if they accept. If it is coming out of goodwill from them, they will not need the visibility and then we can be serving the community without any publicity from them, and the money will go to good use.” (Participant 13, local agency)*

In addition, three participants mentioned requiring consent forms from parents before posting any photos of children they worked with. Other conditions reported include the corporate banners, full branding, guidelines for the activities, and paying customs costs for shipped products.

Only two participants mentioned there were no conditions stipulated by the corporations when supporting the humanitarian organizations:

*“They have never asked us for anything really, I don’t think that there is an advantage to them. They would maybe either like to help or distribute their stock before it expires or maybe it is for self-satisfaction”. (Participant 11, local agency)*

### Perceived benefits to humanitarian organizations

Participants reported a number of benefits from corporate assistance both for them and the populations they served. Corporate funding was seen as reliable and available in comparison to governmental funding, and supported programs that were relevant to the needs of the country and disadvantaged communities. This funding also supported the sustainability of the organizations themselves by paying salaries and helping them grow.

The participants used the term “flexibility” to describe corporate funding, which was much needed for diversifying funding sources to sustain their operations in comparison to international and UN funding which required tedious bureaucratic processes, had rigid requirements and fixed agendas.

*“What makes it more flexible is that we can discuss these requirements. If they (corporations) put certain criteria that we see are not feasible, then we can negotiate to make them more practical and realistic to be achieved. To diversify our source of income, we cannot only rely on UN agencies or EU agencies because they have very clearly set political agendas that change from one year to the other. So we need to diversify the sources to make sure that the needs on the ground are really being covered. Private sector can negotiate more, especially the local ones as they understand the context you are in, because they are in it as well.” (Participant 5, local agency)*

*“They [corporations] are less bureaucratic. So, you can identify a person to talk to and you can have a back-and-forth conversation and that is very helpful in comparison with international agencies who you don’t know and it is a one-way communication process.” (Participant 6, local agency)*

Other notable benefits included establishing long-term relations with corporations which facilitated effective communication and negotiation with key people in corporations, which in turn was described to facilitate the approval process as compared to institutional donors who seem to be tied down with bureaucracy. Uncomplicated logistics and shorter reports were also described cited by many participants as timesaving; and increased visibility for the humanitarian organizations, and increased reach to people through the corporate marketing strategies were also of added value.

*“… the impact of the program, the messages they are reaching people; what is missing is part of monitoring and evaluation, and they are better at it than us, because they have done it all the time as part of their advertising and marketing work, and thus they have access to the general population more than us.” (Participant 2, international agency)*

### Perceived benefits to corporations

All participants mentioned that corporations benefit from increased visibility through providing support to humanitarian organizations serving the public. As explained by one of the participants, corporations map out active and visible humanitarian organizations to partner with, and expect a benefit in return. Many of our participants spoke of the interest the corporations have in corporate social responsibility (CSR), which plays a role in enhancing their reputation, and consequently expanding the marketing of their products by increasing their reach to new consumers.

*“when the private sector comes to the NGO, they want to partner with them, so that when they have audits, they would score high on CSR. And sometimes, even after our trainings and interventions, we ask them to continue, for example with the referral process, and they refuse, because they are really not interested, they just want a stamp of approval to improve their reputation and to gain this accreditation.” (Participant 11, local agency)*

*“They are more interested in the social media visibility:‘let’s show that we are teaching those kids’, ‘let’s show through a small video that we signed an MoU with you to do this and this’. So it is more about PR (public relations) rather than charity.” (Participant 13, local agency)*

### Perceived disadvantages

Three participants stated no disadvantages to receiving money from corporations, while all other participants spoke of several. These disadvantages manifest at the level of ethical considerations and programmatic issues. These include the motive of self-interest behind corporate funding rather than humanitarian outcomes. Other concerns related to the transparency and neutrality of their work, the risk of receiving tainted money whose sources are unknown, and a risk to the humanitarian agency’s name being associated with the corporations of problematic reputations, such as tobacco and alcohol, and to a lesser extent, others such as Nestlé. But the organizations seemed not be able to offer convincing reasons beyond, for example, the potential for having a bad reputation and consequent mistrust from the public.

*“We cannot for example take funds from Nestlé for any program that has to do with child or infant feeding, because we know of the problems that Nestlé has historically in terms of interfering with breastfeeding and child nutrition programs. […] There is always a reputational risk. […] We would be affiliated with them and it might ruin our reputation.” (Participant 2, international agency)*

Other programmatic level disadvantages mentioned include corporations being unaware of local needs, and being interested in tangible results only; unreliable and limited long term funding opportunities; and insufficient funding for full programs. Four participants mentioned that obtaining funds from corporations can be a time-consuming process as it entails back and forth communication, writing long and meticulous proposals that meet the requirements of the funding agencies, especially in case of multiple funders and a detailed budget allocation as well as the lengthy discussions with human resource departments for vetting the source. Post submission, funders may for further documentation and if funded, reports on how the funds were spent are required. Other disadvantages included creating competition among humanitarian organizations and potential conflicts between the recipients of the same fund.

### Perceived influence on the recipients of humanitarian aid

We had not discussed the perceived influence of corporate assistance with recipients of humanitarian aid, however our participants rather shared their own views, which varied. Over a third of our participants told us that they saw no harm to the recipients, and referred to a variety of reasons, including that: i) because corporate funding is not substantial, there is no harm from showing the company’s logo to their recipients and others; ii) the humanitarian organizations design the project in a way that there were no negative impacts on the recipients; iii) the recipients would not have access to social media anyway, so could not see the brands and corporations posted there; iv) that products such as Nestlé or PepsiCo are already available everywhere, so working with the companies would not change anything; or v) recipients do not have the money to buy these products anyway.

*“for example, Pampers and Colgate are giving out products to beneficiaries that are not likely to become their clients soon because these people can’t even afford buying these brands” (Participant 10, local agency)*

*“It does not have an impact because the people we cater for don’t have access to social media to start with……If Pepsi or Coca Cola or Nestlé or whoever—these kids do not relate to the big world, they don’t know who they are. You tell them a name, but they don’t recognize it.” (Participant 13, local agency)*

Three participants even suggested that the humanitarian organizations had a buffering role between the corporations and recipients when designing the interventions, or by advising recipients on healthy living in case of health harming products:

*“…beneficiaries, they are free, you know, I will tell them do not buy things, or do not attach yourself to things that are harmful—I use healthy food and I am aware of [what is a healthy] and I will try to direct these people to stay away from things that will harm them.” (Participant 9, local agency)*

*“It depends on how you design the project as an NGO and how you handle, and not let the influence from the donors negatively impact your beneficiaries. You are at the forefront, at the front, so you want to make sure that you are doing the project right, developmentally right, without any negative by-product […], the way we do the buffer between the donor and the end beneficiary, to make sure they get nothing but the positive benefit that they need.” (Participant 5, local agency)*

Meanwhile, three of the participants acknowledged that there was potential influence on the recipients, which manifests in the form of increased attachment or loyalty to the brand and products.

*“Of course he will be loyal to Pepsi. He is not going to go buy Coca-Cola when Pepsi taught him” (Participant 14, local agency)*

*“Yes they create an emotional attachment to the brand. But what I want to say is that from what I realized, they are always interested to share their videos in other parts of the world, like for example in Europe to show how they helped in other places of the world.” (Participant 10, local agency)*

However, a few of the participants relayed their belief that corporate assistance had a positive influence on recipients because of the opportunities provided by humanitarian aid to develop the recipients’ skills, increase their employment opportunities, or connect with role models.

*“Well, this is difficult to answer, but from my subjective point of view, there is a positive impact. For example, an NGO has connections with artists, celebrities in the competition that we had to develop skills for youth. Those who got the awards, got them through a celebration, and it was attended by celebrities, and so they were given the opportunity to interact with a piano player and movie producer…” (Participant 4, international agency)*

## Discussion

This study took a critical stance on an emerging topic: corporate assistance to humanitarian organizations. In particular, it examined the presence or absence of ethical and governance guidelines regarding corporate-humanitarian arrangements inside humanitarian organizations of all sizes, and how staff implementing humanitarian programs and services within these organizations describe the ethics, costs, and benefits of these arrangements.

Hotho and Girschick (2019) argue that the literature has not sufficiently addressed the various ways by which firms engage in humanitarian assistance and the complications that ensue from such interaction [41]. The political economy of aid assistance during crises is layered with dilemmas in every region of the world, and when crises are compounding, these dilemmas are amplified. Refugees and Lebanese people in Lebanon who, because of the multiple crises which have recently hit the country, have become disadvantaged and experience multiple vulnerabilities. These crises in the absence of a strong supportive state have created a convenient opportunity for corporations to step in and aid local humanitarian organizations. Humanitarian organizations, over the past decades, took over the role of the government in service provision, particularly during the 1975–1990 civil war, and recently with the Syrian refugee crisis [42].

The humanitarian organizations included in our study reported receiving funding, in-kind donations and services mainly from the food, beverage and pharmaceutical sectors. While corporate contributions to and engagement with larger international organizations are ongoing for protracted crises, such as response to and governance of a decade-long refugee crisis, corporate-sponsored humanitarian aid was also implemented directly and episodically in the aftermath of more rapid onset crises, in the immediate aftermath of the Beirut port blast, or on special occasions such as religious holidays, with donations during *Adha Eid*.

Overall, our participants noted that the process for receiving funding and other incentives from corporations was less labor intensive and bureaucratic than applying for funding to other sources, such as international and UN organizations. This was perceived as a positive aspect of corporate funding, together with the increased visibility for the humanitarian organizations and the possibility to broaden their reach, using the existing markets which corporations work in. Meanwhile, corporations had specific conditions in exchange for the support provided, including showing logos and brands on social media. This reflects the fact that marketing is a central incentive in these arrangements, which was reported as acceptable and even justifiable by some of our interview participants. Whatever agendas, aims or motives funding sources bear, their support comes at a time when humanitarian funding is low globally, and humanitarian organizations are therefore not in a position to be fastidious about the sources of their income.

This study has shown that corporations distribute aid to small, local civil society organizations, and corporate funding is also channeled through multilateral organizations (i.e. UNHCR) that in turn, provide direct services to humanitarian organizations, with the government’s consent. With the decrease in the capacity of those international organizations to effectively respond to humanitarian crises, “non-traditional actors such as corporations, particularly multinational corporations, become increasingly involved in the delivery of humanitarian assistance” [41 p.202]. This aspect of corporate influence on humanitarian assistance was not captured by our study, but merits further investigations.

This study contributes to the scarce body of knowledge pertaining to the ethical dilemmas that corporate assistance to humanitarian aid poses and the issues that arise from it. In our study, both parties seemed to benefit from the current arrangements they have: humanitarian organizations sustain their operations and are able to intervene or provide services in place of a weak state, while corporations allocate their funds or provide low cost services and establish relations with reputable organizations. While humanitarian organizations have a mandate to operate in neutral capacities, the source of financial flows and conditionalities (whether formal or informal) that are attached to them complicate the notion of neutrality, especially if support comes from profit making entities. Marks (2019) argues that close relations between civil society organizations and corporations can lead to staff altering their views of the industry; the humanitarian agencies thus becoming an “unintentional proxy for the industry providing them with credibility enhancement” [43 p.72]. Baur and Schmitz (2012) describe this ‘cooptation by the industry’ as problematic as it creates dependency, and challenges the humanitarian organizations’ ability to contest corporate requests, while becoming accountable to the corporation as is the case with the local ones in the study, meanwhile introducing market-driven logic into the non-for-profit sector [44]. The engagement of firms in the delivery of humanitarian assistance is often contingent on expected returns, threatening the sustainability of these interactions between corporations and humanitarian organizations [41]. Corporations have vast resources, managerial efficiency, technical expertise, and access to finance [45], while humanitarian organizations have expertise and knowledge of the needs of the field, are mission driven, and are better able to reach disadvantaged populations [46]. The two entities are incompatible at an organizational level as they have varying competencies, missions, goals, and accountability. Corporations are profit-oriented do not want to be political actors with responsibility to the people, and do not engage in assessments of needs [47].

This context poses a question about the type of collaboration and the quality of the work implemented by the humanitarian organizations, and the extent of true “partnership”—a term used repeatedly by our participants to describe their relationships with the corporations, and which reflects the point our participants made about acceptable requirements by the corporations assisting them. Partnerships are widely promoted by UN organizations, as explained earlier. The UN Global Compact was for example mentioned by one of our participants for justifying their work with companies. ‘Partnerships’ or ‘public–private partnerships’ therefore became an acceptable term and used to include a range of different activities and processes for interaction with humanitarian organizations [22,48]. Global rules of trade often favor corporations and the powerful countries where they are based, which makes the corporations the decision makers, capable of approaching and choosing who to collaborate with and support [26,41]. Humanitarian organizations included in our study were, and for the most part, unaware of the possibility of conflicts of interest that their collaborations with the private sector poses. No participant mentioned any problems that necessitated placing a hold on or halting their already established relationships with the corporations which have provided such assistance.

Beyond these issues related to the humanitarian organizations themselves, corporations have an interest in penetrating new markets, as well as improving their image if they support reputable humanitarian organizations. Corporations create partnership models which address short-term funding gaps, but which come with long-term health-harming outcomes among vulnerable populations. Several organizations in this study were supported to do their work with disadvantaged youth and refugees, which illustrates this point about funders’ interest in creating new markets and loyalties to their brand. The agenda described above was not totally unknown to the humanitarian organizations; many participants associated this assistance to the self-interest of companies in marketing their brands, under the guise of CSR as a “business opportunity”^44^. By engaging with humanitarian aid, corporations seek to enhance their “legitimacy and reputation in the eyes of their stakeholders, consumers, and governments” [41 p.209]. Refugees and recipients of humanitarian aid may become instrumentalized—they are in vulnerable situations and are so prone to develop positive emotional responses to the brands and logos they are exposed to [43].

The benefits mentioned by our participants seemed nevertheless to outweigh any potential harms to the people they assist. There is currently little done by the organizations we interviewed to address the issues mentioned above. The World Association of NGO (WANGO) Code of Ethics and Conduct relates to funding and partnerships. Only 12 such organizations working in Lebanon signed up to the Code, none of which were amongst our participants [49]. The principles cited in the Code of WANGO, and which are relevant to our study, are those of transparency, independence, accountability, and funding consistent with the mission of organization, and free of coercion. These did not seem to be observed by corporations as they are not familiar with the conduct of humanitarian work and have a different set of work ethics [41]. The guidelines our participating humanitarian organizations were limited to Codes of Conduct for their staff, and pertained to their interactions with the people they serve, and professional conflicts of interest. The questions of conflicts of interest and undue influence were not considered by the humanitarian organizations when applying for funding, but some mentioned that they would not receive money from corporations with contradicting values. None of our participants seemed to have, or at least were aware of, any guidelines in their organizations that may help with the decision making for collaborations with external organizations of any type. The reference to certain values which they believe in seemed to serve as a firewall against harmful interaction. The obvious health harming industries, such as tobacco and alcohol, were cited in six interviews as not being acceptable as sources of humanitarian assistance; yet despite these value systems and processes put in place, a number of participants reported that their organizations were ‘flexible’ and would consider assistance from the industry because of their need for financial support, and described it as an added value.

Lack of guidelines or policies for humanitarian organizations may lead to incidents of exploitation/misconduct by corporations or the humanitarian organizations themselves. These are important points to consider in future research, since corporate involvement in humanitarian work may translate in corruption, through bias and preferential treatment.

There are a few limitations to our study that are worth noting. First, our study took place during the COVID-19 pandemic and after the 2020 Beirut Port blast, which was a chaotic and overwhelming working environment for many. We consequently had limited access to potential interviewees, who for the most part, were not accessible due to competing responsibilities. These exceptional circumstances however, provided ripe grounds for the study and helped us better understand corporate funding in a context where funding is scarce and urgently needed. Another limitation is related to the recruitment of our participants–with a topic of a rather sensitive nature, humanitarian organizations might prefer not to disclose their sources of funding or mode of work, in general, and they may want to avoid engaging in a contentious topic, such as that of corporate funding. It also might be that many saw the topic as not of a concern to them because they have not been recipients of such funding. Moreover, the fact that none of our participants had signed the WANGO Code of Ethics and Conduct may be indicative of transparency as being a low priority for those organizations, making them less likely to discuss information on their internal workings. This is a fact that should not be underscored in the humanitarian scene in Lebanon [50].

This study has implications for future research. It would be valuable to extend the work to other contexts and policy environments where humanitarian organizations and corporations are active, and how the presence or absence of regulation on corporate influence and conflicts of interest influences their behavior. Such research can take place in other countries, in Europe for example, that have received influx of refugees, or in countries that were part of a humanitarian crisis, such as most recently Ukraine. Recent evidence points out to the Ukrainian crisis as a likely ripe ground for exploitation by corporations [51,52]. It is also worth understanding the perspectives of, and impacts on, the people themselves who are on the receiving end of humanitarian aid. This will provide a more in depth understanding of benefits and costs and of potential short- or long-term harms, exploitation and misconduct. Finally, it is important to undergo a thorough review of available written guidelines and Codes of Conduct that might exist and guide humanitarian organizations. This could be performed as part of a comprehensive review to understand the landscape of humanitarian assistance and aid. This review can inform practice and contribute to the development of a charter or guideline to promote best practices. Such guidelines can in turn be used in training on commercial determinants of health in the context of humanitarian assistance. Finally, it is the responsibility of the international and local organizations to sign up to guidelines that can regulate and mitigate their engagement with corporations to minimize any ensuing potential harms.
